# Differential privacy in collaborative filtering recommender systems: a review

**DOI:** 10.3389/fdata.2023.1249997

**Published:** 2023-10-12

**Authors:** Peter Müllner, Elisabeth Lex, Markus Schedl, Dominik Kowald

**Affiliations:** ^1^Know-Center Gmbh, Graz, Austria; ^2^Institute of Interactive Systems and Data Science, Graz University of Technology, Graz, Austria; ^3^Institute of Computational Perception, Johannes Kepler University Linz, Linz, Austria; ^4^Linz Institute of Technology, Linz, Austria

**Keywords:** differential privacy, collaborative filtering, recommender systems, accuracy-privacy trade-off, review

## Abstract

State-of-the-art recommender systems produce high-quality recommendations to support users in finding relevant content. However, through the utilization of users' data for generating recommendations, recommender systems threaten users' privacy. To alleviate this threat, often, differential privacy is used to protect users' data via adding random noise. This, however, leads to a substantial drop in recommendation quality. Therefore, several approaches aim to improve this trade-off between accuracy and user privacy. In this work, we first overview threats to user privacy in recommender systems, followed by a brief introduction to the differential privacy framework that can protect users' privacy. Subsequently, we review recommendation approaches that apply differential privacy, and we highlight research that improves the trade-off between recommendation quality and user privacy. Finally, we discuss open issues, e.g., considering the relation between privacy and fairness, and the users' different needs for privacy. With this review, we hope to provide other researchers an overview of the ways in which differential privacy has been applied to state-of-the-art collaborative filtering recommender systems.

## 1. Introduction

Several previous research works have revealed multiple privacy threats for users in recommender systems. For example, the disclosure of users' private data to untrusted third parties (Calandrino et al., 2011), or the inference of users' sensitive attributes, such as gender or age (Zhang et al., 2023). Similarly, also the users themselves care more about their privacy in recommender systems (Herbert et al., 2021). For these reasons, privacy-enhancing techniques have been applied, most prominently *differential privacy (DP)* (Dwork, 2008). DP injects random noise into the recommender system and formally guarantees a certain degree of privacy. However, through this random noise, the quality of the recommendations suffers (Berkovsky et al., 2012). Many works aim to address this trade-off between recommendation quality and user privacy via carefully applying DP in specific ways. Friedman et al. (2016) show that in case of matrix factorization, DP can be applied to three different parts of the recommender system: (i) to the input of the recommender system, (ii) within the training process of the model, and (iii) to the model after training. However, a concise overview of works with respect to these three categories does not exist yet.

Therefore, in the paper at hand, we address this gap and identify 26 papers from relevant venues that deal with DP in collaborative filtering recommender systems. We briefly review these 26 papers and make two key observations about the state-of-the-art. Firstly, the vast majority of works use datasets from the same non-sensitive domain, i.e., movies. Secondly, research on applying DP after model training is scarce. Finally, we discuss our findings and present two open questions that may be relevant for future research: *How does applying DP impact fairness?* and *How to quantify the user's perceived privacy?*

Our work is structured as follows: In Section 2, we present threats to the privacy of users in recommender systems and additionally, introduce the DP framework. In Section 3, we precisely outline our methodology for obtaining the set of 26 relevant papers. In Section 4, we review these papers and group them into three groups according to the way in which they apply DP. In Section 5, we discuss our findings and propose open issues that we identified.

## 2. Background

In recent years, users of recommender systems have shown increasing concerns with respect to keeping their data private (Herbert et al., 2021). In fact, several research works (Bilge et al., 2013; Jeckmans et al., 2013; Friedman et al., 2015; Beigi and Liu, 2020; Majeed and Lee, 2020; Himeur et al., 2022) have revealed multiple privacy threats, for example, the inadvertent disclosure of users' interaction data, or the inference of users' sensitive attributes (e.g., gender, age).

Typically, a recommender system utilizes historic interaction data to generate recommendations. Ramakrishnan et al. (2001) show that in *k* nearest neighbors recommender systems, the recommendations could disclose the interaction data of the neighbors, i.e., users, whose interaction data is utilized to generate the recommendations. Similarly, Calandrino et al. (2011) inject fake users to make the recommendations more likely to disclose the neighbors' interaction data, and also, they can infer users' interaction data based on the public outputs of a recommender system, e.g., public interaction data or public product reviews. Furthermore, Hashemi et al. (2022) and Xin et al. (2023) aim to learn user behavior via observing many recommendations and, in this way, can disclose parts of a user's interaction data. Weinsberg et al. (2012) show that an adversary could infer sensitive attributes, in this case, gender, based on a user's interaction data. Their attack relies on a classifier that leverages a small set of training examples to learn the correlation between a user's preferences and gender. Likewise, Ganhör et al. (2022) show that recommender systems based on autoencoder architectures are vulnerable to infer the user's gender from the latent user representation. The authors also propose an adversarial training regime to mitigate this problem. Similarly, also Zhang et al. (2023) infer the age and gender of users in a federated learning recommender system. In summary, many of a user's sensitive attributes can be inferred via thoroughly analyzing the user's digital footprint (e.g., the behavior in a recommender system or social media platform) (Kosinski et al., 2013).

Overall, the utilization of users' interaction data for generating recommendations poses a privacy risk for users. Therefore, privacy-enhancing techniques, such as homomorphic encryption (Gentry, 2009), federated learning (McMahan et al., 2017), or most prominently, *differential privacy (DP)* (Dwork, 2008) have been applied to protect users' privacy. Specifically, DP is applied via injecting noise into the recommender system. This ensures that the recommender system uses noisy data instead of the real data. For example, an additive mechanism samples random noise from the Laplace or Gaussian distribution and adds it to the users' rating data (Dwork and Roth, 2014). Alternatively, the randomized responses mechanism flips a fair coin, which decides whether to use the real data or random data, and this way, ensures DP (Warner, 1965; Dwork and Roth, 2014). Overall, the degree of noise that is used is defined by the parameter ϵ, i.e., the privacy budget. Intuitively, the smaller the ϵ-value is, the better the privacy, but the stronger the expected accuracy drop. Therefore, choosing ϵ is non-trivial and depends on the specific use case (Dwork, 2008).

## 3. Review methodology

To conduct our review, we chose relevant conferences in the field, i.e., ACM SIGIR, TheWebConf, ACM KDD, IJCAI, ACM CIKM, and ACM RecSys and journals, i.e., TOIS, TIST, UMUAI, and TKDE. Adopting a keyword-based search, we identified relevant publications in the proceedings via querying the full-texts for “differential privacy” and “recommender system”, “recommend”, “recommendation”, or “recommender”. We manually checked the resulting papers for their relevance and retrieved 16 publications. In addition, we conducted a literature search on Google Scholar using the same keywords and procedure, which resulted in 10 publications. Overall, we considered 26 publications in the paper at hand.

## 4. Recommender systems with differential privacy

According to Friedman et al. (2016), DP can be applied via (i) adding noise to the input of a collaborative filtering-based recommender system, e.g., the user data or other user representations, (ii) adding noise to the training process of the model, i.e., the model updates, or (iii) adding noise to the model after training, i.e., to the resulting latent factors. In Table 1, we group the selected publications into these three categories.

**Table 1:** Overview of the reviewed 26 publications.

		**DP applied to**		
**References**	**Domain(s)**	**User represent**.	**Model updates**	**After training**
Long et al. (2023)	Location	•		
Müllner et al. (2023)	Movies, Music, Books, Social	•		
Neera et al. (2023)	Movies, Jokes, Dating	•		
Wang et al. (2023)	Movies, Music		•	
Chai et al. (2022)	Movies, Location	•		
Chen et al. (2022)	Movies, Music, Books	•		
Jiang et al. (2022)	Movies, Music, Location, Groceries		•	
Liu et al. (2022)	Social		•
Ning et al. (2022)	Movies		•
Ran et al. (2022)	Movies, Music			•
Ren et al. (2022)	Social	•		
Wu et al. (2022)	Advertisement	•		
Li et al. (2021)	Movies, Dating		•
Minto et al. (2021)	Movies		•
Zhang et al. (2021)	Movies	•		•
Chen et al. (2020)	Location	•		
Gao et al. (2020)	Movies, Smartphone	•		
Ma et al. (2019)	Health		•
Meng et al. (2018)	Social		•
Shin et al. (2018)	Movies, Dating		•
Liu et al. (2017)	Movies	•	
Yang et al. (2017)	Movies	•		
Li et al. (2016)	Movies	•		
Hua et al. (2015)	Movies		•	•
Zhu et al. (2013)	Movies	•	
Zhao et al. (2011)	Movies	•		

We mark whether DP is applied to the user representation, to the model updates, or after training. Domain(s) refers to the domain(s) in which the recommendations are evaluated. We sort the publications with respect to recency.

### 4.1. Differential privacy applied to the user representation

In collaborative filtering recommender systems, the input to the system is typically given by interaction or rating data. However, more complex user representations exist, e.g., neural-based user embeddings.

Chen et al. (2020) protect POI (point of interest) interaction data of users, e.g., a user visited a restaurant, with DP. Specifically, they use this data to privately calculate POI features, i.e., the number of visitors per restaurant, which are subsequently used for generating recommendations instead of the DP-protected interaction data. This way, they can increase recommendation accuracy. Similarly, Long et al. (2023) use DP to recommend POIs, but in a decentralized fashion. A central server collects public data to train a recommendation model and to privately identify groups of similar users. DP is used for privately calculating user-user similarities. Then, users locally use information from similar users, which leads to a better trade-off between recommendation quality and privacy than comparable approaches.

Liu et al. (2017) add noise to users' rating data and to the user-user covariance matrix to ensure DP of a KNN-based recommender system. They show that this leads to better privacy than in case only the covariance matrix is protected via DP. Besides revealing users' rating data, an attacker could also aim to infer sensitive attributes (e.g., gender) of the users. Therefore, Chai et al. (2022) propose an obfuscation model to protect gender information. After applying this obfuscation model, users protect their data via DP and send it to a central server. Yang et al. (2017) use the Johnson-Lindenstrauss transform (Blocki et al., 2012), i.e., they ensure DP via multiplying the original interaction matrix with a random matrix. Using this protected matrix, their approach guarantees differential privacy and also can even generate more accurate recommendations than a non-private approach. Neera et al. (2023) underline that adding Laplacian noise can lead to “unrealistic” rating values, i.e., outside the rating range, and through this, recommendation accuracy can drop severely. Therefore, they bound the noisy ratings to a “realistic” value range without harming DP. Plus, they use a Gaussian mixture model to estimate and then remove noise in the recommendation process to keep recommendation accuracy.

Cross-domain recommendation models can increase recommendation accuracy in the target domain by exploiting data from multiple source domains. To protect user privacy when data from the source domain is made available to the target domain, Chen et al. (2022) use the Johnson-Lindenstrauss transform. Due to the high sparsity of the rating matrix, they employ a variant that performs better when applied to sparse matrices (Ailon and Chazelle, 2009). Ren et al. (2022) utilize data from different social network platforms to generate recommendations and apply DP to the user attributes and the connections in the social network graphs. Plus, they apply a variant of DP to protect textual data (Fernandes et al., 2019). Moreover, to increase the click-through rate for recommended advertisements, Wu et al. (2022) leverage user interaction data from multiple platforms. First, user embeddings are generated per platform and then protected with DP. Second, the recommender system collects and aggregates a user's DP-protected embeddings across platforms and then applies DP again to the aggregated user embedding. According to the authors, applying DP after aggregation allows for smaller noise levels when applying DP to the per-platform user embeddings, which results in higher accuracy. Typically, many users use a variety of different online platforms. Therefore, Li et al. (2016) leverage these multiple data sources per user to increase recommendation accuracy. Specifically, they combine DP-protected item-item similarities from dataset *B* as auxiliary data that helps to generate more accurate recommendations for users in dataset *A* (cf. Zhao et al., 2011).

Gao et al. (2020) compute item-item similarities by using DP-protected user interaction data. With these item-item similarities, users can locally generate recommendations on their own devices, therefore not harming their privacy. The item-based KNN recommender system proposed by Zhu et al. (2013) utilizes DP in two ways: First, they randomly rearrange the most similar neighbors to foster privacy. Second, they measure how the item-item similarity changes if a specific user interaction was not present, and with this, they add the necessary level of noise to the users' interactions. This way, recommendation accuracy can be better preserved than with approaches that apply the same level of noise to all user interactions. For user-based KNN, Müllner et al. (2023) identify neighbors that can be reused for many recommendations. This way, only a small set of users are used as neighbors for many recommendations and need to be protected with DP. Many users, however, are only rarely utilized as neighbors and therefore do not need to be protected with DP. Overall, this yields more accurate recommendations than in case DP needs to be applied to all users.

### 4.2. Differential privacy applied to the model updates

Some recommender systems do not process user data and create user representations on a central server, instead, they compute the model updates, i.e., gradients, locally on their users' device. Then, the recommender system collects these gradients to adapt its recommendation model. To prohibit the leakage of user data through these gradients (Bagdasaryan et al., 2020), DP can be applied.

For example, Hua et al. (2015) add noise to the gradients of the recommendation model to ensure DP. However, due to the sparsity of the gradients, the application of DP can be ineffective and information about what items have been rated by the user can be disclosed. To address this problem, Shin et al. (2018) use DP to mask whether a user appears in the dataset. Also, they formally show that the noise added to the gradients hinders a fast convergence of the recommendation model, and in this way, increases the training time. Therefore, they introduce a stabilization factor to enable better training of the recommendation model. Wang et al. (2023) propose a recommender system that uses a special DP-mechanism (Zhao et al., 2020) to simultaneously protect the rating values and the set of items that is rated by a user. The DP-protected item-vectors are then send to a central server, which performs dimensionality reduction to reduce the accuracy drop (cf. Shin et al., 2018). In Minto et al. (2021), users receive a global model from a central server and, then, compute their respective updates locally. These updates are protected via DP, before being sent back to the server. Plus, the number of updates per user are restricted to further improve privacy. Moreover, the authors highlight that high-dimensional gradients can negatively impact the recommendation quality, as they are especially prone to higher sparsity (cf. Hua et al., 2015; Shin et al., 2018). When DP is applied, the gradients become denser since noise is added to the entire gradient, including the zero-entries. This, in practice, leads to additional communication overhead, since all non-zero-entries need to be transmitted (Ning et al., 2022). Therefore, Ning et al. only add noise to the non-zero gradients. This way, the communication overhead is reduced; however, DP cannot be guaranteed anymore.

Jiang et al. (2022) reduce the accuracy drop via an adaptive DP mechanism that depends on the number of training steps. Intuitively, after many training steps, the model fine-tunes its predictions and the gradients need to be measured more accurately than during the beginning of the model training. Thus, they add more noise in the beginning and less noise in the end of the training process. This yields more accurate recommendations than a static DP mechanism that always adds the same level of noise. Li et al. (2021) also use noisy model updates to ensure DP. They observe that noise can lead to large values for the user embeddings, which increases the sensitivity and therefore also the level of noise that is required to ensure DP. To foster recommendation quality, they map the user embeddings to a certain range, which bounds the sensitivity and requires less noise. Liu et al. (2022) leverage user interactions and social connections to generate recommendations via a federated graph neural network. To ensure DP, they add noise to the gradients that are sent to a central server. However, gradients with different magnitudes have different sensitivities (cf. Li et al., 2021), and thus, need a different level of noise to ensure DP. Therefore, they fit the noise level to the gradient magnitudes to satisfy DP, but also, to preserve recommendation accuracy.

Ma et al. (2019) employ federated tensor factorization in the health domain. A global model is distributed to hospitals, which locally update the model based on their data. To protect privacy, a variant of DP is applied to the model updates, which are subsequently sent to the global server to adapt the global model. Meng et al. (2018) randomly divide users' ratings into non-sensitive and sensitive ratings. For sensitive ratings, they apply more noise than for non-sensitive ratings. With this, their approach can preserve higher recommendation accuracy than in case the same noise level is used for sensitive and non-sensitive data.

### 4.3. Differential privacy applied after training

Only few works apply DP to the recommendation model after training. In case of a matrix factorization approach, noise can be added to the learned user- and item-vectors to ensure DP. Our selected publications (see Section 3) do not include any works that apply DP exclusively to the model after training. Nevertheless, we describe works that apply DP to the user representation or the model updates, but also after training.

For example, Hua et al. (2015) consider a matrix factorization model, where the model sends item-vectors back to the users and this way, users' data can get leaked. To prohibit this, Hua et al. perturb the model's objective function after training via adding noise to the latent item-vectors. Similarly, Ran et al. (2022) also use DP to prohibit data leakage through the item-vectors that are sent to the users. Specifically, a trusted recommender system generates a matrix factorization model. Instead of publishing the item-vectors of this model, they learn new item-vectors on the DP-protected user-vectors. Through this, they can minimize the noise that is introduced and thus, can improve recommendation accuracy over comparable approaches. Zhang et al. (2021) apply DP to the user representation and also, to the model after training. Specifically, they use a polynomial approximation of the model's loss function to efficiently compute the sensitivity of the dataset and, accordingly, adapt the level of noise that is added to the loss function.

## 5. Summary and open questions

**Figure 1:**
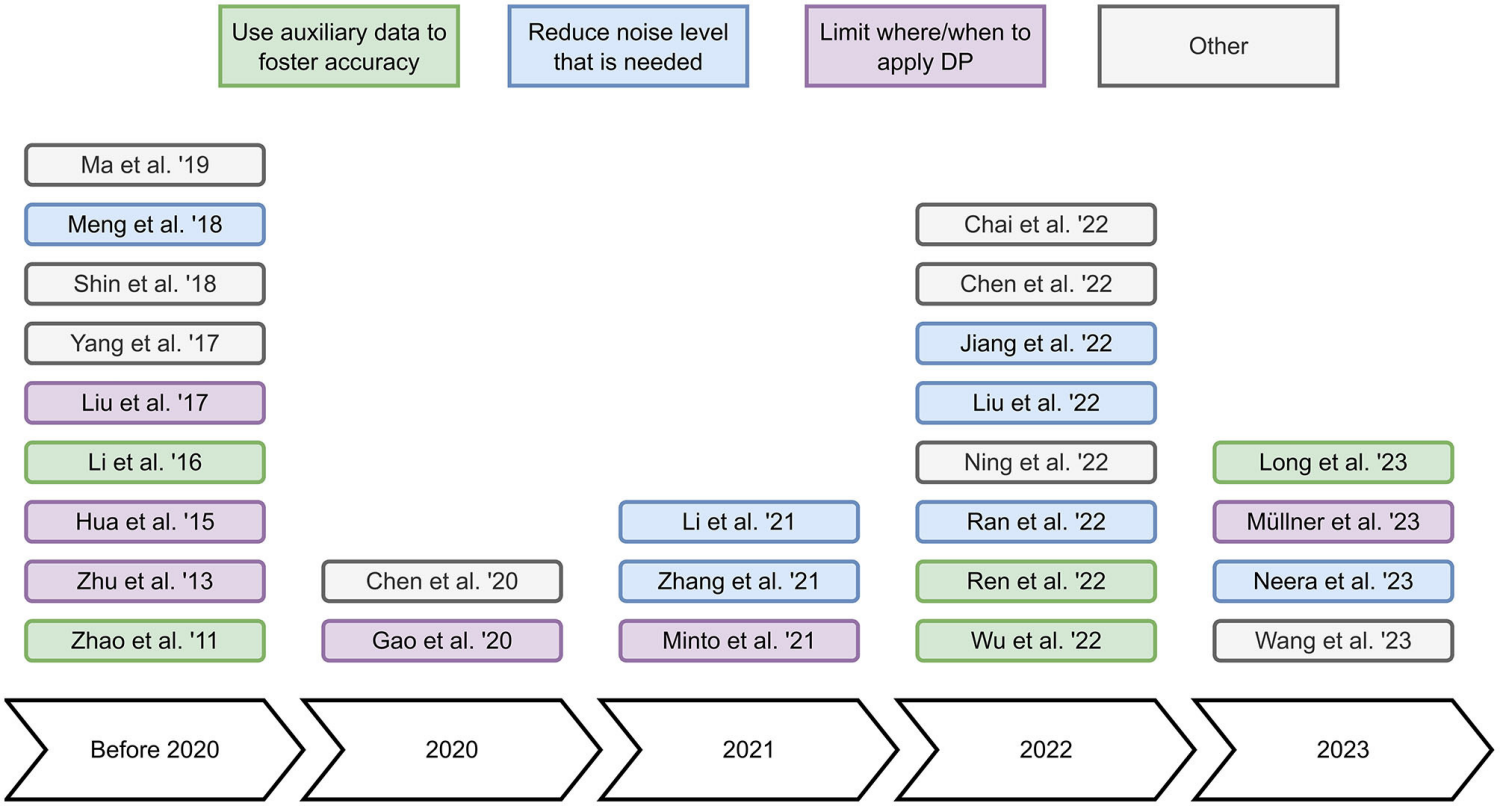
Overview of the main concepts of the reviewed papers. *Use auxiliary data to foster accuracy* refers to the incorporation of data from other domains, datasets or users to increase recommendation accuracy. *Reduce noise level that is needed* refers to designing recommender systems that require a minimal amount of noise to ensure DP. *Limit where/when to apply DP* refers to carefully minimizing the application of DP. *Other* refers to approaches that do not fit into the previous categories.

In this review, we investigate research works that apply DP to collaborative filtering recommender systems. We identify 26 relevant works and categorize these based on how they apply DP, i.e., to the user representation, to the model updates, or to the model after training (see Table 1). In addition, we briefly summarize these relevant works to obtain a broad overview of the state-of-the-art. Furthermore, we identify the main concepts of the relevant works in Figure 1 to help readers to understand in which diverse ways the reviewed papers apply DP to improve the accuracy-privacy trade-off. Our main findings from reviewing the discussed literature are two-fold: (i) The majority of works use datasets from the same non-sensitive domain, i.e., movies, and (ii) applying DP to the model after training seems to be an understudied topic.

Many research works use datasets from the movie domain, which, in general, does not include sensitive data. For research on DP in collaborative filtering recommender systems, however, datasets from sensitive domains may be better suited to resemble real-world privacy threats well. For example, datasets from the health, finance, or job domain. Moreover, the majority of research focuses on either applying DP to the user representation or to the model updates. Research on applying DP to the model after training is scarce, and therefore, this opens up the possibility of future work to fill this gap.

Our review of relevant work allows to grasp the state-of-the-art and to identify the following open research questions:

*Q1: How does applying DP impact fairness?* Dwork et al. (2012) and Zemel et al. (2013) suggest that in theory, privacy can lead to fairness and fairness can lead to privacy. The reason is that for both, a user's data shall be hidden, either to ensure privacy or to prohibit discrimination based on this data. However, in practice, correlations in private data can still lead to unfairness (Ekstrand et al., 2018; Agarwal, 2020). Only recently, Yang et al. (2023) and Sun et al. (2023) investigate the connection between privacy and fairness in recommender systems. For example, Sun et al. (2023) use DP-protected information to re-rank the items in the recommendation list and in this way, increase a more fair exposure of items. Nonetheless, the impact of DP on fairness remains an understudied topic.

*Q2: How to quantify the user's perceived privacy?* Users perceive privacy differently, e.g., some users tolerate disclosing their gender, while others refuse to do this (Joshaghani et al., 2018). This perceived privacy depends on many factors, e.g., context or situational factors (Knijnenburg and Kobsa, 2013; Mehdy et al., 2021). However, measuring users' perceived privacy is hard and is usually done via questionnaires (Knijnenburg and Kobsa, 2013). This is in stark contrast to how privacy is measured in the DP framework, i.e., via quantifying to what extent the data impacts the output of the recommender system. Therefore, developing methods to better quantify users' privacy is an important future research avenue.

## Author contributions

PM: literature analysis, conceptualization, and writing. MS: conceptualization and writing. EL and DK: conceptualization, writing, and supervision. All authors contributed to the article and approved the submitted version.
